# Sleep quality among cardiac patients on follow up at Jimma Medical Center, southwestern Ethiopia

**DOI:** 10.5935/1984-0063.20190154

**Published:** 2021

**Authors:** Yared Getahun, Wondu Reta Demissie, Hiwot Amare

**Affiliations:** 1 Jimma University, Institute of Health, Faculty of Medical Science, School of Medicine, Department of Internal Medicine - Jimma - Oromia - Ethiopia.; 2 Jimma University, Institute of Health, Faculty of Medical Science, School of Medicine, Department of Biomedical Sciences - Jimma - Oromia - Ethiopia.

**Keywords:** Sleep Quality, PSQI, Cardiac Patients, JMC, Ethiopia, Associated Factors

## Abstract

**Introduction:**

Chronic heart failure is associated with changes in sleep pattern and affects quality of sleep among patients with heart failure. Poor sleep has a negative impact on the patients’ quality of life, furthermore it compromises cognition and one’s self-care practice. Though, factors affecting sleep among heart failure patients have been investigated in developed world, there are limited studies in regards to it in developing countries like Ethiopia.

**Objective:**

The aim of the study is to assess the level of sleep quality and associated factors among heart failure patients who are on follow up at Jimma Medical Center (JMC).

**Material and Methods:**

Hospital based cross sectional study was employed among the total sample of 111 chronic heart failure patients admitted to medical ward and having follow up at cardiac center of JMC. The data was collected from April 1 - April 30, 2019 through face-to-face interview by using structured questionnaire. Pittsburg Sleep Quality Index (PSQI) was applied to assess sleep quality. PSQI score <5 refers to good sleep quality. Data was checked, cleaned for possible errors, entered into Epidata version 4.3.1 and ﬁnally exported to SPSS version 20 for further analysis. Appropriate statistical analyses (cross tabulation and logistic regression) were applied. A p value less than 0.05 was used to declare statistical signiﬁcance.

**Results:**

The mean age was 49±14 that ranges from 20-89 years by which majority of them 34 (30.6%) belongs to interval age of 35-44 years. The mean of PSQI score was 4.9±2.79 that ranges from 1-17 scores. Majority of the cardiac patients [69(62.2%)] were considered as having good sleep status (PSQI score <5) while the remaining 42 (37.8%) were considered as poor sleepers with PSQI score >5. Two variables (age of patients more than 65 years and presence of comorbidity) were identiﬁed as associated factors with poor sleep quality having P-value less than 0.05 and speciﬁc AOR with 95%CI of 4.087 (2.013-8.612) and 3.042 (1.074-8.619), respectively.

**Conclusion:**

Poor sleep quality is common in heart failure patients. Age >65 years and comorbidities are predictors of poor sleep quality in these patients.

## INTRODUCTION

Heart failure (HF) is a growing problem worldwide, with almost 23 million people affected. It is estimated that by 2030 the prevalence will increase to 25% from current estimates^1^. The prevalence of HF rises exponentially with age and affects 4% to 8% of people older than 65 years^2^. The overall prevalence of HF is thought to be increasing and putting it as a major public health issue, with a prevalence of over 5.8 million in the USA, and over 23 million worldwide, and rising^3,4^.

Sleep is an important aspect of maintaining the body’s circadian rhythm^5,6^. Inadequate sleep contributes to heart disease, diabetes, depression, falls, accidents, impaired cognition, and a poor quality of life^7^. While normal aging changes interfere with the quality of sleep, other disease conditions and medications used by older adults also compromise sleep patterns^8,9^.

Disturbed sleep is among the most frequent health complaints that physicians encounter in their careers. More than one-half of adults in the United States experience at least intermittent sleep disturbance, and only 30% of adult Americans report consistently obtaining a sufficient amount of sleep^10^.

Chronic heart failure is an important cause of high levels of sleep disturbance and disorder^11^. Poor sleep is among the most frequently reported symptoms of patients with heart failure^12^. The proportion of poor sleepers among patients with HF is among the highest in chronic diseases^13^. Dyspnea and fatigue as common symptoms of this disease, significantly increase the chance of being a bad sleeper^14^.

The contributors of sleep disturbances among people with HF appear to be multi-factorial and complex. There is growing recognition that sleep-related breathing disorders, including Cheyne-Stokes respiration with central sleep apnea (CSR-CSA) and obstructive sleep apnea (OSA), are common in people with HF, with a range of 10% to 60%^15^. They have been shown consistently to cause sleep disruption due to repetitive arousals from sleep. It is evident that CSR-CSA is more likely to occur in patients with HF with left ventricular ejection fractions less than 45%, whereas OSA may be an independent risk factor to the development of HF and a consequence of HF^16^. Other factors that contribute to poor sleep in heart failure patients include demographic characteristics, pathophysiology of HF, comorbid health problems, symptoms of HF, medications and primary sleep disorders^17^.

Epidemiological studies are required and encouraged in regards/aspects of the common health problems of HF patients. However, unfortunately, there are limited studies that conducted on the sleep quality of HF patients in Ethiopian and at setting (the recent study reported that one third of hypertensive patients were poor sleepers)^18^. But the source population doesn’t represent the heart failure patients. Thus, it is worth to undertake studies related to sleep disturbance and associated factors among heart failure patients in Ethiopia and the present study aimed to determine sleep quality and its associated factors.

## MATERIAL AND METHODS

The study was conducted in JMC from April 01-30/2019 among already diagnosed heart failure patients. Hospital based cross-sectional study was employed among randomly selected 111 samples of cardiac patients who admitted to medical ward and at follow up in cardiac center for treatment as the hospital guideline and protocol from an estimated 500 heart failure patients visiting the institution per year. The sample was enrolled from heart failure patients who had at least 6-month duration since visited the institution for treatment and/or follow up while heart failure patients with concomitant mental disorders and who take any medication were excluded from the study as they affect sleep quality.

JMC is one of the teaching referral hospitals of the country located in Jimma town, southwest of Ethiopia at about 350 Kilometers from the capital, Addis Ababa. JMC serves millions of patients dwelling in the catchments area of southwest part of the country. It has the cardiac unit that provides both inpatient and outpatient service for patients with CVDs.

Quality of sleep was assessed by using Pittsburg Sleep Quality Index (PSQI) tool, which has seven components: subjective sleep quality (Component 1), sleep latency (Component 2), sleep duration (Component 3), habitual sleep efficiency (Component 4), sleep disturbances (Component 5), use of sleep medicine (Component 6) and daytime dysfunction (Component 7)^19^. The result from all these seven components were calculated (added together to generate PSQI scores) and if the sum of the components yields PSQI score of less than 5, it is considered as respondents were good sleeper and PSQI score >5 refers to poor sleeper. PSQI was validated in the country and used as the best tool to assess sleep quality among different population of the country as well as the specific study setting (JMC)^20^.

The collected data was checked for completeness by principal investigator, and then data was entered into Epidata 4.3.1 version and finally exported to SPSS version 20 for further analysis. Descriptive statistics were performed by percentages, means, medians, standard deviations and ranges to describe finding. Cross tabulation and logistic regression were applied to determine the association between outcome variable (sleep status) and predictors. A *p*-value of 0.05 was declared as statistically significant. Ethical clearance was obtained from ethical review committee of Jimma University, Institute of Health Sciences. Both informed verbal and written consent was obtained. Participants’ confidentiality, equity of services and interests was ensured.

## RESULTS

### Socio-demographic status of cardiac patients

A total of 111 cardiac patients were enrolled with the mean age of 49±14 years that ranges from 20-89 years where majority of them 34 (30.6%) belongs to the age interval of 35-44 years, males 60 (54.1%), married 62(55.9%), private employee 29(26.1%), complete primary school 29(26.1%), Oromo 57 (51.4%), Muslims 60 (54.1%) and dwellers of urban 59 (53.2%), as detailed in Table 1.

**Table 1:** Socio-demographic characteristics of cardiac patients at JMC, 2019.

Variables	Categories	Frequency	Percentage (%)
Residence	Urban	59	53.2
	Rural	52	46.8
Sex	Male	60	54.1
	Female	51	45.9
Age in years	<34	17	15.3
	35-44	34	30.6
	45-54	22	19.8
	55-64	18	16.2
	>65	20	18.0
Educational status	Can't read and write	22	19.8
	Only read and write	8	7.2
	Primary school	29	26.1
	Secondary school	26	23.4
	Higher education	26	23.4
Religious status	Muslim	60	54.1
	Orthodox	32	28.8
	Protestant	16	14.4
	Catholic	3	2.7
Ethnicity	Oromo	57	51.4
	Amhara	17	15.3
	Tigre	9	8.1
	SNNP	28	25.2
Occupation	Farmer	21	18.9
	House wife	27	25.2
	Private	29	26.1
	Public	16	14.4
	Unemployed	7	6.3
	Retired	10	9.0
Marital status	Married	62	55.9
	Widowed	12	10.8
	Divorced	20	18.0
	Single	17	15.3

### Cardiac status of patients

Specific types of cardiac diseases were discriminated as congestive heart disease (CHD) 37 (33.3%), ischemic heart disease (IHD) 26 (23.4%), hypertension 20 (18.0%), rheumatic heart disease (RHD) 10 (9.0%) and others 18 (16.2%) like ischemic heart disease. Majority of the cardiac disease patients 55 (49.5%) had also other comorbidities like diabetes mellitus, thyroid disorders, stroke and renal problems. Majority of the cardiac disease patients 73 (65.8%) developed disease within five years and their heart status belong to stage two (39.6%) according to New York heart association (NYHA). The mean duration of illness was 5.1±4.9 years that range from 0.5-30 years as seen in supplement file in Table 5.

**Table 5:** Cardiac status of patients at JMC, 2019

Variables	Categories	Frequency	Percentage(%)
Presence of comorbidity	Yes	55	49.5
	No	56	50.5
Duration of disease	≤5years	73	65.8
	>5years	38	34.2
Typeof disease	CHD	37	33.3
	RHD	10	9.0
	IHD	26	23.4
	HTN	20	18.0
	Other	18	16.2
NYHA classification	NYHAI	26	23.4
	NYHAII	44	39.6
	NYHAIII	19	17.1
	NYHAIV	22	19.8

### Substance use among cardiac patients

Current use of substance within the last three month was assessed among cardiac patients and majority of them were nonsmokers 98 (88.3%), non-drinkers 87 (78.4%), non-chewers 70 (73.1%), but majority of them 88 (79.3%) drink coffee/tea (supplement file in Table 6).

**Table 6:** Substance use among cardiac patients at JMC, 2019

Variables	Categories	Frequency	Percentage(%)
Smoking status	Smoke	13	11.7
	Not smoke	98	88.3
Alcoholic status	Drinker	24	21.6
	Not drinker	87	78.4
Chewings tatus	Chewer	41	36.9
	Not chewer	70	63.1
Coffee/tea drinking	Drinking	88	79.3
	Not drinking status	23	20.7

### Sleep status of cardiac patients

Pittsburgh Sleep Quality Index (PSQI) was used to assess the quality and patterns of sleep among cardiac patients. Cardiac patients subjectively allocated their sleep status as very good 65 (58.6%), fairly good 31 (27.9%), fairly bad 10 (9.0%) and very bad 5 (4.5%) for component one and other components were summarized in Table 2. The mean of duration of time spent on the bed by cardiac patients was 8.7±1.5 hours that range from 2-14 hours but the mean duration of actual sleep time was 7.9±2.4 hours that range from 2-12 hours. The mean of duration of time taken to fall asleep was 29.72±19.3 minutes that range from 5-90 minutes as explained in detail in Table 2.

**Table 2:** Sleep status among cardiac patients at JMC, 2019.

Variables	Categories	Frequency	Percentage (%)
Component I (subjective perceived sleep quality)	Very good (Score 0)	65	58.6
	Fairly good (Score 1)	31	27.9
	Fairly bad (Score 2)	10	9.0
	Very bad (Score 3)	5	4.5
Component II (sleep latency) Time taken to fall asleep plus 5A score.	Very good (Score 0)	9	8.1
	Fairly good (Score 1)	64	57.7
	Fairly bad (Score 2)	32	28.8
	Very bad (Score 3)	6	5.4
Component III (Actual sleep duration in hours)	>7 hours (Score 0)	75	67.6
	6-7 hours (Score 1)	14	12.6
	5-6 hours (Score 2)	18	16.2
	<5 hours (Score 3)	4	3.6
Component IV (Habitual sleep efficiency) (total # of hrs. asleep) x 100 (total # of hrs. in bed)	>85% (Very good) (Score 0)	74	66.7
	75-84% (Fairly good) (Score 1)	22	19.8
	65-74% (Fairly bad) (Score 2)	9	8.1
	>65% (Very bad) (Score 3)	6	5.4
Component V (sleep disturbances) Sum of Scores #5b to #5j (0=0; 1-9=1; 10-18=2; 19-27=3)	Very good (Score 0)	0	0
	Fairly good (Score 1)	82	73.9
	Fairly bad (Score 2)	29	26.1
	Very bad (Score 3)	0	0
Component VI (use of sleep medicine)	Not at all ( 0)	100	90.1
	Less than once a week (1)	8	7.2
	Once to twice a week (2)	3	2.7
	Three or more times a week (3)	0	0
Component VII (Daytime dysfunction)	Very good (Score 0)	60	54.1
	Fairly good (Score 1)	41	36.9
	Fairly bad (Score 2)	10	9.0
	Very bad (Score 3)	0	0

**Figure 1:**
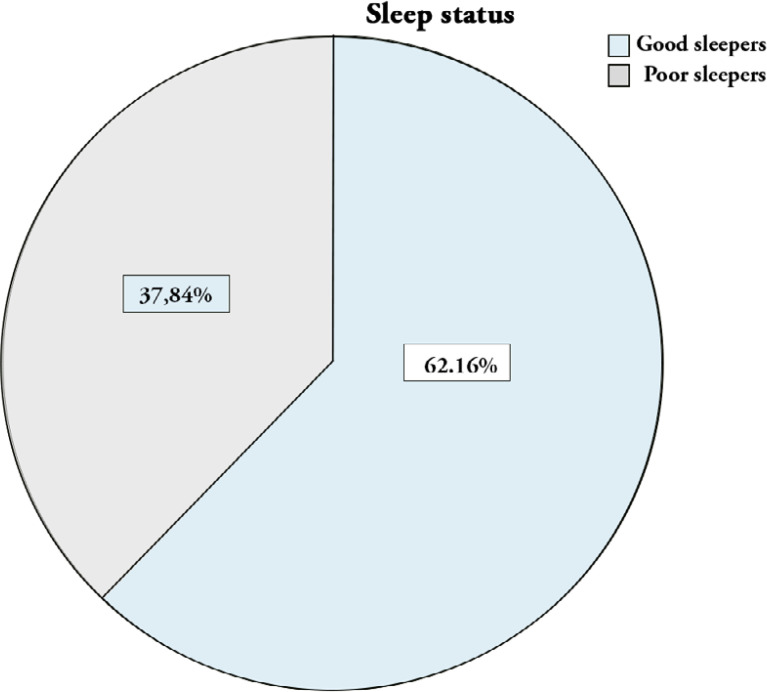
Status of sleep among cardiac patients at JMC, 2019

Finally, the global PSQI score was computed for all patients by adding results of all components together. The mean of PSQI score was 4.9±2.79 that ranges from 1-17 scores. In nutshell, majority of the cardiac patients [69 (62.5%)] were considered as having good sleep status with the value of PSQI score less than five, while the remaining 42 (37.8%) were considered as poor sleepers with PSQI score five, or more than five (online supplementary file in Figure 1).

### Factors associated with sleep status among cardiac patients

To identify factors associated with sleep status, logistic regression analysis was applied. In the bivariate analysis, the candidate variables having *p*-value<0.25 were selected for the final model. Accordingly, about seven variables (sex, age, educational status, BMI, smoking, alcohol drinking and presence of comorbidity) were identified as the expected factors associated with sleep status with their specific chi-square, COR with 95%CI and *p*-values as explained in Table 3 in details.

**Table 3:** Association of sleep status and other variables by bivariate logistic regression analysis among cardiac patients at JMC, 2019.

Dichotomous variables	Categories	Status of Sleep			COR(95% CI)	P- value	X^2^
		Good, No (%)	Poor, Nº (%)	Total, Nº (%)			
Residence	Urban	37(33.4)	22(19.8)	59(53.2)	1		
	Rural	32(28.8)	22(19.8)	52(46.8)	1.05(0.4-2.2)	0.899	0.016
Sex	Male	33(29.7)	27(24.4)	60(54.1)	1.96(0.8-4.3)	0.093*	
	Female	36(32.4)	15(13.5)	51(45.9)	1		2.84
Age in years	≤34	13(11.7)	4(3.6)	17(15.3)	1	0.006*	17.3
	35-44	17(15.3)	17(15.3)	34(30.6)	3.2(0.28-5.2)	0.07*	
	45-54	16(14.4)	6(5.4)	22(19.8)	1.2(1.6-39)	0.79	
	55-64	16(14.4)	2(1.8)	18(16.2)	0.4(0.06-2.5)	0.34	
	≥65	7(6.3)	13(11.7)	20(18.0)	6(1.4-25)	0.01*	
Educational status	Can't read and write	10(9.0)	12(10.8)	22(19.8)	1.9(0.6-6.0)	0.26	7.8
	Only read and write	6(5.4)	2(1.8)	8(7.2)	0.53(0.09-3.1)	0.49	
	Primary school	23(20.7)	6(5.4)	29(26.1)	0.41(0.12-1.3)	0.15*	
	Secondary school	14(12.6)	12(11.8)	26(23.4)	1.37(0.4-4.1)	0.57	
	Higher education	16(14.4)	10(9.0)	26(23.4)	1	0.12*	
Religious status	Muslim	36(32.4)	24(21.6)	60(54.1)	1.3(0.1-15)	0.81	
	Orthodox	21(18.9)	11(9.9)	32(28.8)	1.048(0.08-12)	0.97	
	Protestant	10(9.0)	6(5.4)	16(14.4)	1.2(0.08-16)	0.89	0.31
	Catholic	2(1.8)	1(0.9)	3(2.7)	1	0.95	
Ethnicity	Oromo	34(30.6)	23(20.7)	57(51.4)	1	0.85	0.78
	Amhara	12(10.8)	5(4.5)	17(15.3)	0.6(0.19-1.9)	0.41	
	Tigre	6(5.4)	3(2.7)	9(8.1)	0.7(0.16-3.2)	0.69	
	SNNP	17(15.3)	11(9.9)	28(25.2)	0.95(0.3-2.4)	0.92	
Occupation	Farmer	14(12.6)	7(6.3)	21(18.9)	1	0.82	
	House wife	19(17.1)	9(8.1)	27(25.2)	0.94(0.28-3.1)	0.93	
	Private	15(13.5)	14(12.6)	29(26.1)	1.86(0.58-5.9)	0.29	2.17
	Public	10(9.0)	6(5.4)	16(14.4)	1.2(0.3-4.6)	0.79	
	Unemployed	5(4.5)	2(1.8)	7(6.3)	0.8(0.12-5.2)	0.81	
	Retired	6(5.4)	4(3.6)	10(9.0)	1.3(0.28-6.3)	0.71	
Marital status	Married	40(36.1)	22(19.8)	62(55.9)	1	0.95	
	Widowed	7(6.3)	5(4.5)	12(10.8)	1.29(0.3-4.5)	0.68	0.34
	Divorced	12(11.8)	8(7.2)	20(18.0)	1.21(0.4-3.4)	0.71	
	Single	10(9.0)	7(6.3)	17(15.3)	1.27(0.4-3.8)	0.66	
BMI	Normal	45(40.5)	34(30.6)	79(71.2)	1	0.18*	3.87
	Under weight	6(5.4)	1(0.9)	7(6.3)	0.22(0.002-1.9)	0.17*	
	Overweight	18(16.2)	7(6.3)	25(22.5)	0.5(0.19-1.3)	0.18*	
Smoking status	Smoke	5(4.5)	8(7.2)	13(11.7)	3(0.9-9.9)	0.070*	3.3
	Not smoke	64(57.7)	34(30.6)	98(88.3)	1		
Alcoholic status	Drinker	12(10.8)	12(10.8)	24(21.6)	1.9(0.7-4.7	0.16*	1.88
	Not drinker	57(51.4)	30(27.0)	87(78.4)	1		
Chewing status	Chewer	24(21.6)	17(15.3)	41(36.9)	1.2(0.57-2.8)	0.54	0.36
	Not chewer	45(40.5)	25(22.6)	70(63.1)	1		
Coffee/tea drinking status	Drinking	55(49.6)	33(29.7)	88(79.3)	0.9(0.3-2.3)	0.88	0.021
	Not drinking	14(12.6)	9(8.1)	23(20.7)	1		
Presence of comorbidity	Yes	29(26.2)	26(23.4)	55(49.5)	2.2(1.02-4.9)	0.044*	4.1
	No	40(36.1)	16(14.4)	56(50.5)	1		
Duration of disease	<5 years	44(39.6)	29(26.1)	73(65.8)	1.2(0.5-2.8)	0.57	0.32
	>5 years	25(22.5)	13(11.7)	38(34.2)	1		
Type of disease	CHD	23(20.7)	14(12.6)	37(33.3)	0.76(0.24-2.3)	0.63	3.4
	RHD	4(3.6)	6(5.4)	10(9.0)	1.87(0.39-9)	0.43	
	IHD	18(16.2)	8(7.2)	26(23.4)	0.55(0.15-1.9)	0.35	
	HTN	14(12.6)	6(5.4)	20(18.0)	0.53(0.14-2)	0.35	
	Other	10(9.0)	8(7.2)	18(16.2)	1	0.51	
NYHA classification	NYHA I	17(15.3)	9(8.1)	26(23.4)	1	0.96	0.29
	NYHA II	27(24.3)	17(15.3)	44(39.6)	1.18(0.4-3.2)	0.73	
	NYHA III	11(9.9)	8(7.2)	19(17.1)	1.37(0.4-4.6)	0.6	

COR: Crude Odd Ratio; X2: Chi square;

* Candidate for final mode (p-value <0.25).

Further, multivariate analysis (binary logistic regression with enter methods) was applied to identify the main predictors. Finally, two variables (age of patients more than 65 years and presence of comorbidity) were identified as the factors associated with sleep status among cardiac patients with *p*-value less than 0.05 and specific AOR (95% CI). Patients who had additional comorbidity were three times more likely to become poor sleepers than those who had no comorbidity and patients greater than 65 years were also about four times likely to be poor sleepers with specific AOR (95% CI) of 4.087(2.013-8.612) and 3.042(1.074-8.619) respectively as explained in Table 4 in details.

**Table 4:** Association of sleep status and other variables by multivariate logistic regression analysis among cardiac patients at JMC, 2019.

Variables candidate in multivariate logistic regression	AOR	95% C.I	P-value
Sex (male)	2.466	0.8-7.2	0.099
Age classification (>65 years)	4.087	2.0-8.6	0.004
Education (Can't read and write)	1.113	0.2-5.9	0.900
BMI class (normal)	0.229	0.02-2.6	0.232
Smoking (yes)	3.137	0.6-16.0	0.170
Alcohol (yes)	0.739	0.18-2.9	0.671
Additional comorbidity (yes)	3.042	1.1-8.6	0.036

## DISCUSSION

Among the total enrolled 111 cardiac patients, the mean age was 49±14 that ranges from 20-89 years by which majority of them 34 (30.6%) belong to interval age of 35-44 years. The present result was comparable with previous study done in the setting by Abebe et al.^21^ who reported the mean age of 49±16 years and comparable baseline characteristics among heart failure patients.

Majority of the cardiac disease patients 73 (65.8%) developed disease within five years with dominant NYHA stage two (39.6%) which was also supported by study of Chen et al.^22^, Wang et al.^23^ and Santos et al.^14^ who also reported NYHA class II dominance (72%), (63.4%) and (50.5%), respectively. The mean duration of illness was 5.1±4.87 years that range from 0.5-30 years.

The Pittsburgh Sleep Quality Index (PSQI) was used to assess the quality and patterns of sleep among cardiac patients, as it is an effective instrument used to differentiate “poor” from “good” sleep by measuring seven domains (subjective sleep quality, sleep latency, sleep duration, habitual sleep efficiency, sleep disturbances, use of sleep medication and daytime dysfunction) over the last month. The tool has internal consistency and a reliability coefficient (Cronbach’s alpha) of 0.83 for its seven components. A global sum of “5” or greater indicates a “poor” sleeper. The mean of PSQI score was 4.9±2.79 that ranges from 1-17 scores. Majority of the cardiac patients [69 (62.2%)] were considered as having good sleep status with the value of PSQI score less than five, while the rest 42 (37.8%) were considered as poor sleepers with PSQI score five or more than five. The present finding of mean PSQI score of 4.9 was within the global PSQI score range of 3-20^24^.

The present study revealed less magnitude of poor sleep quality (37.8%) among cardiac patient in relative to previous studies: Abebe et al.^21^ (81.65%), Wang et al.^23^ (81.2%), Moradi et al.^24^ (79%), Chen et al.^22^ (74.4%) and Santos et al.^14^ (68.5) who reported high magnitude of poor sleep quality among heart patients probably due to age differences [all reported mean age greater than the present mean age (48.9, 74.0, 73.1, 67.8 and 57.8, respectively)], socio-demographic differences and variation in season of data collection period. But, the prevalence of poor sleep quality in present finding was in harmony with the study of Wong and Fielding^25^ who reported the prevalence of poor sleep quality/insomnia of (39.4%) among total population.

The mean of PSQI score of 4.37±2.64 in the present study was not comparable with findings of Abebe et al.^21^, Santos et al.^14^, Moradi et al.^24^ and Wang et al.^23^ who reported higher mean of PSQI score of 9.23, 10.8, 9.8 and 10.0, respectively; probably due to study population differences especially age difference (the present participant were relatively less elders/low mean age) as studies confirmed disturbance of sleep quality as age advances^26,27^. The study also revealed that status of sleep quality affected by different age groups. The prevalence of insomnia was 44.4% for the elderly group and 31.4% for the younger group as compared and reported by Gau et al.^28^.

In general, the prevalence of sleep disturbances among heart failure patients was within the range of 35-70% and the present finding revealed the rate of poor sleep quality (37.8%) that also supported by other studies who reported the rate of poor sleep quality within these ranges^17,29,30^.

The mean of duration of time spent on the bed by cardiac patients was 8.7±1.5 hours that range from 2-14 hours while mean duration of actual sleep time was 7.9±2.4 hours that range from 2-12 hours. This finding was relatively higher than mean daily duration of sleep of 5.6 hours and 6 hours reported by Moradi et al.^24^ and Santos et al.^14^, respectively, probably due to socio-demographic difference.

The mean of duration of time taken to fall asleep was 29.72±19.3 minutes that range from 5-90 minutes, which was in line with the study of Santos et al.^14^ and Zeighami and Shahparian^31^ who reported the mean duration of sleep latency of 42±36 minutes and 42 minutes, respectively.

Cardiac patients were subjectively allocated their sleep status as very good 65 (58.6%), fairly good 31 (27.9%), fairly bad 10 (9.0%) and very bad 5 (4.5%) for component I of PSQI tool. But, this pattern of allocation by component I where patients with score 0 (very good) dominant was not in line with studies of Abebe et al.^21^, Santos et al.^14^ and Wang et al.^23^, who reported allocation of more patients in score 2 (53%, 47% and 31%), respectively.

When cardiac patients were assessed for sleep status by component II of PSQI tool, they were belong to fairly good 64 (57.7%) who fall asleep within 16-30 minutes, fairly bad 32 (28.8%) who fall asleep within 31-60 minutes, very good 9 (8.1%) who fall asleep within 15 minutes and very bad 6 (5.4%) who fall asleep after 60 minutes where this pattern was also supported by study of Abebe et al.^21^ who reported majority of patients (35.3%) with score 1 by component II of PSQI tool.

According to assessment done by PSQI tool component III, majority of cardiac patients [75 (67.6%) were belong to score 0 (very good) having more than 7 hours of actual sleeping per night followed by score 2/fairly bad 18 (16.2%), score 1/fairly good 14 (12.6%) and score 2/fairly bad 4 (3.6%). This pattern was also supported by study of Abebe et al.^21^ who reported allocation of majority of patients (43.5%) with score 0 by component III PSQI tool.

According to assessment done by PSQI tool component IV, the scores of habitual sleep efficiency of cardiac patients were allocated to score 0 (very good) 74 (66.7%), score 1/fairly good 22 (19.8%), score 2/fairly bad 9 (8.1%) and score 3/very bad 6 (5.4%) where this pattern was also supported by study of Abebe et al.^21^ who reported majority of patients (29.5%) with score 0 using component IV PSQI tool.

When cardiac patients were assessed with component V of PSQI tool (sleep disturbances), they were only classified to score 1/fairly good 82 (73.9%) and score 2/fairly bad 29 (26.2%) while there was no any patients with score 0 and 3. However, this pattern was not supported by study of Abebe et al.^21^ who also reported the prevalence of 2.9% and 4.3 % in score 0 and 3, respectively, by component V PSQI tool.

According to assessment performed by PSQI tool component VI (use of sleep medicine), majority of cardiac patients 100 (90.1%) were not used medication to induce sleep within the past one month while the rest use medication to induce sleep within the past one month at frequency of less than once a week 8 (7.2%), once to twice a week 3 (2.7%). This pattern was also in harmony with the studies of Santos et al.^14^ and Abebe et al.^21^ who revealed that 89.5% and 95.7% of the study participants didn’t use medication to sleep respectively. But, this finding was against the study of Moradi et al.^24^ who reported that only about 61.5% of participants didn’t use medication to induce sleep.

According to assessment by PSQI tool component VII (daytime dysfunction), the scores of daytime dysfunction of cardiac patients were belong to score 0 (very good) 60 (54.1%), score 1/fairly good 41 (36.9%), score 2/fairly bad 10 (9.0%) and no patients with score 3/very bad. This pattern was against the study of Abebe et al.^21^ who reported that 20.9% of participants allocated to score 3 probably due to study population differences especially age difference (the present participant were relatively less elders/low mean age).

In nutshell, when all components of PSQI were evaluated for screening sleep quality, worst scale of poor sleep (score 3) was only discerned by component I-IV with the prevalence of 4.5%, 5.4%, 3.6% and 5.4%, respectively. But, the prevalence of score 3 was 0% if assessed by other PSQI components of V-VII.

In bivariate analysis of logistic regression, the correlation of poor sleep status was observed with about seven variables (sex (male) 1.96 (0.8-4.3), age (>65 years) 6 (1.4-25), educational status (illiterate) 1.9 (0.6-6.0), BMI (overweight) 0.5 (0.19-1.3), smoking (yes) 3 (0.9-9.9), alcohol drinking (yes) 1.9 (0.7-4.7 and presence of comorbidity (yes) 2.2 (1.02-4.9)) having *p*-value less than 0.25 and specific COR with 95%CI as explained in Table 3 in detail. However, only two variables (age of patients more than 65 years and presence of comorbidity) were identified as associated factors with poor sleep quality having *p*-value less than 0.05 and specific AOR with 95%CI of 4.087 (2.013-8.612) and 3.042 (1.074-8.619), respectively, as detailed in Table 4 from the results of multivariate analysis of logistic regression.

The possible interpretation and inference was drawn as; patients who had additional comorbidity were three times more likely to become poor sleepers than those who had no comorbidity and patients greater than 65 years were also about four times likely to be poor sleepers. This finding was also supported by other studies^23,24,28,31,32^ where different predictors (comorbidity, age, educational status, smoking, income and BMI) were determined to affect sleep quality.

## CONCLUSION

Despite, the present finding revealed less prevalence (37.8%) of poor sleep quality in relative to studies conducted so far among heart failure patients for possible reasons, it is common in heart failure patients, especially among aged patients of >65 years and with comorbidities.
